# Feminization and masculinization of western mosquitofish (*Gambusia affinis*) observed in rivers impacted by municipal wastewaters

**DOI:** 10.1038/srep20884

**Published:** 2016-02-15

**Authors:** Guo-Yong Huang, You-Sheng Liu, Xiao-Wen Chen, Yan-Qiu Liang, Shuang-Shuang Liu, Yuan-Yuan Yang, Li-Xin Hu, Wen-Jun Shi, Fei Tian, Jian-Liang Zhao, Jun Chen, Guang-Guo Ying

**Affiliations:** 1 State Key Laboratory of Organic Geochemistry, CAS Centre for Pearl River Delta Environmental Pollution and Control Research, Guangzhou Institute of Geochemistry, Chinese Academy of Sciences, Guangzhou 510640, China

## Abstract

Municipal wastewaters have been known to contain various estrogens and androgens. Little is known about the joint action of these chemicals from wastewaters on fishes in the aquatic environment. The objectives of this study were to investigate the estrogenic and/or androgenic effects in wild mosquitofish (*Gambusia affinis*) of two effluent-impacted rivers in South China by determining morphological changes and hepatic mRNA expression levels of relevant genes such as vitellogenin (Vtg), estrogen receptor (ERα) and androgen receptors (ARα and ARβ), and to assess the linkages of those morphological changes and hepatic mRNA expression levels to the chemical concentrations measured by *in vitro* bioassays and chemical analysis. The results showed a significant induction of Vtg and ERα mRNA in the livers of the males and a gonopodium-like anal fin in the females collected at the majority of sites. Redundancy analysis and Pearson correlation analysis showed that the chemical concentrations obtained by *in vitro* bioassays and chemical analysis had significant correlations with some of the endpoints for the estrogenic and/or androgenic effects in mosquitofish. The findings from this study indicate that the estrogens and androgens present in the two rivers could cause the observed estrogenic and androgenic effects in mosquitofish.

Endocrine disrupting compounds (EDCs) are exogenous substances that can potentially impair the reproductive functions of organisms by interacting with their endocrine systems^1^,^2^,^3^. Municipal wastewaters have been known to contain various EDCs such as natural and synthetic steroids, which could have the potential to mimic the action of hormones^1^,^4^,^5^. Discharge of municipal wastewaters into the aquatic environment may lead to endocrine disrupting effects in aquatic organisms like fish^6^. Many field studies have focused on the estrogenic effects of EDCs in municipal wastewaters in fish, and fish feminization was reported in UK rivers downstream of effluent discharge points, with observed abnormal development of the gonads, reduced secondary sexual characteristics, and increases in vitellogenin (Vtg) protein and/or mRNA expression levels in fish^6^,^7^. Municipal wastewaters also contain various androgenic compounds^4^,^5^, but the potential of municipal effluents to elicit androgenic responses has not been well studied^8^,^9^. However, field studies on the androgenic effects due to pulp mill effluents showed male-biased sex ratios, masculinized anal fin development in female fish, reduced embryos in females, and increased androgen receptor (AR) mRNA expression in males^10^,^11^,^12^. Municipal wastewaters may contain various estrogens and androgens, but rarely has the simultaneous assessment of estrogenic and androgenic effects in organisms been studied^9^,^13^. Moreover, many aquatic environments in China were heavily polluted by various effluents including municipal wastewaters, but the toxic effects including endocrine disrupting effects in the field were limited^13^,^14^. Therefore, endocrine disrupting effects due to discharge of municipal wastewaters need to be investigated further.

Both field and laboratory studies have shown the value of Vtg protein and/or mRNA as broadly accepted indicators of exposure to estrogens in male and juvenile fish^7^,^13^,^15^. Estrogens are nuclear hormone receptor ligands that bind directly to their cognate estrogen receptors (ERs), triggering a cascade of biochemical reactions that eventually lead to the intended effects including Vtg mRNA expression^16^,^17^. In contrast, androgens exert their effects by acting on androgen receptors (ARs). The transcriptional levels of ERs and ARs can be regulated by EDCs^15^,^16^,^18^. Thus ERα and AR mRNA expression could be used for assessing estrogenic and androgenic effects on fish, respectively.

Mosquitofish has been regarded as a sentinel species in the study of EDCs in the aquatic environment owing to distinguishable hormone-dependent sexual dimorphism^2^. Western mosquitofish (*Gambusia affinis*) are widely distributed in the aquatic environments of South China, and they exhibit strong site fidelity. Both juvenile male and female mosquitofish have an anal fin that is the same in structure. However, mature adult males have a modified anal fin called a gonopodium that is derived from the elongation and modification of anal fin rays 3, 4, and 5 under the control of endogenous androgens. Under exposure to exogenous estrogens, a decrease in gonopodial characteristics (feminization) of male mosquitofish has been observed^19^. Furthermore, the anal fin of female mosquitofish exposed exogenously to androgens can be induced to develop into a gonopodium-like structure (masculinization). The development of the anal fin in mosquitofish is accompanied by the modification of the hemal spines on the 14th, 15th, and 16th vertebrae^19^. The modification of the hemal spines has been used as an end point to determine endocrine disrupting effects in mosquitofish^13^,^14^,^19^

The purpose of this study was to assess the co-occurrence of feminization and masculinization in wild mosquitofish of two rivers impacted by municipal wastewaters in South China by determining morphological changes and relevant gene expression levels. In addition, both *in vitro* yeast-based assays (yeast estrogen screen (YES) and yeast androgen screen (YAS)) and chemical analysis were also performed to determine both estrogenic and androgenic activity as well as representative estrogenic and androgenic compounds in the rivers.

## Results

### Concentrations of estrogenic and androgenic compounds

**Figure 1:**
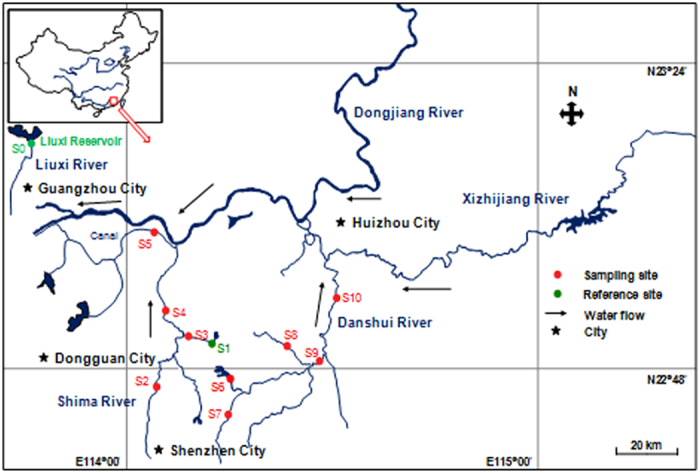
Map showing locations of the sampling sites in the Shima and Danshui Rivers, South China. The red circles represent the sampling sites. The map was created using Adobe Illustrator software.

For steroid estrogens, the average detection frequency of estrone (E1) was the highest (52%), followed by 17β-estradiol (E2) (43%) and diethylstilbestrol (DES) (13%) in all sampling sites of the two rivers Shima River and Danshui River (Fig. 1; Table 1). The detected maximum concentrations for E1, E2 and DES in surface waters were 32.0 ng/L, 3.7 ng/L, and 22.0 ng/L, respectively. 17α-Ethynyl estradiol (EE2) was below the limit of quantification (LOQ) in all water samples. Three xenoestrogens (bisphenol-A (BPA), 4-nonylphenol (4-NP), and 4-t-octylphenol (4-t-OP)) were detected at every sampling site at concentrations of several ng/L to tens of μg/L in surface waters, with maximum concentrations up to 28900 ng/L, 10900 ng/L and 408 ng/L, respectively. Among the 14 target androgens, nine target androgens (androsta-1,4-diene-3,17-dione (ADD), androsterone (ADR), 4-androstene-3,17-dione (AED), 17α-boldenone (17α-BOL), 17β-boldenone (17β-BOL), 5α-dihydrotestosterone (5α-DHT), epi-androsterone (EADR), testosterone (T), and 17β-trenbolone (17β-TBL)) were detected in water samples (Table 1). ADD and AED were the most frequently detected compounds with the detection frequency of >80%.

In addition, both E2 equivalent (EEQ) and DHT equivalent (DEQ) in each water sample were calculated using the relative potencies and environmental concentrations of the chemical concentrations based on the addition model (Table 1). The maximum calculated EEQ and DEQ values (i.e. CEEQ and CDEQ) were 16.0 ng/L and 2280 ng/L, respectively.

### Estrogenic and androgenic activities by *in vitro* bioassays

The estrogenic activity of water samples measured by YES bioassay (MEEQ) ranged from not detected (3% of samples) to 82.6 ng/L (Table 1). The average estrogenic activity of all water samples was 12.6 ng EEQ/L. The androgenic activity of water samples measured by YAS bioassay (MDEQ) varied in the range of not detected to 46.0 ng DHT/L, with a mean of 9.29 ng DHT/L. However, only 42% of all collected water samples were above the limit of detection for the YAS bioassay (Table 1).

### Morphological characteristics of mosquitofish

**Figure 2:**
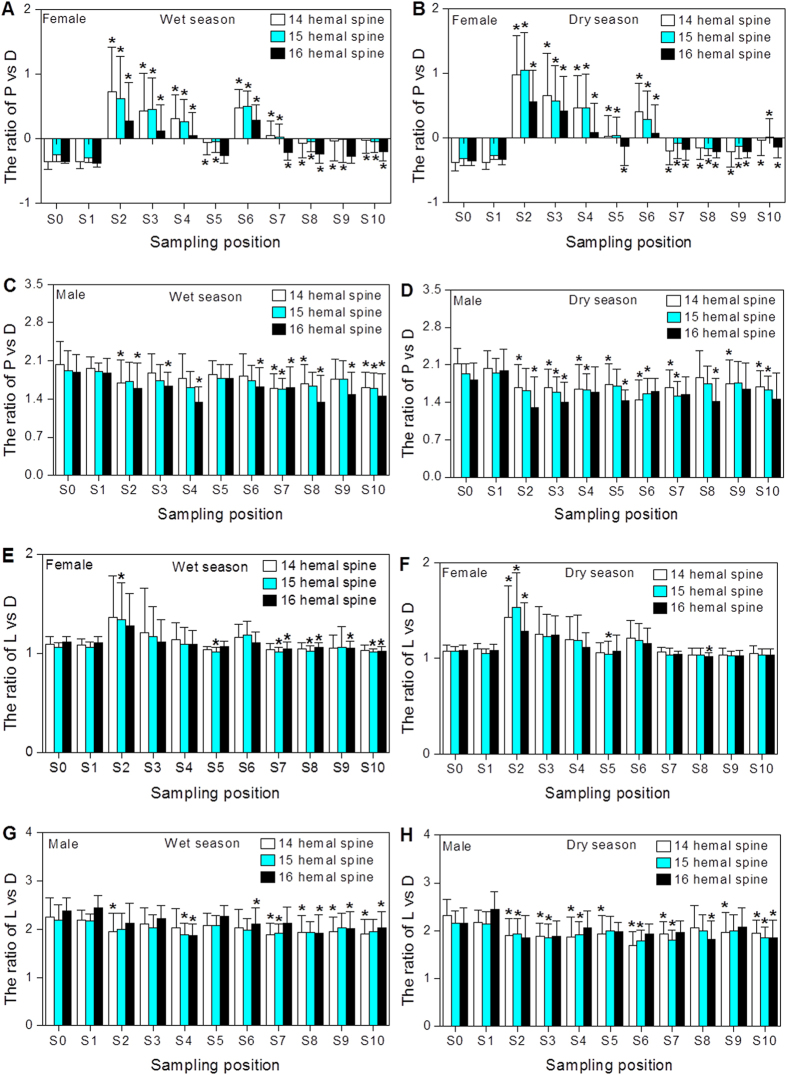
Skeletal morphological characteristics based on the 14th, 15th, and 16th hemal spines for female and male mosquitofish from the Shima and Danshui Rivers as well as the reference site. P: perpendicular distance from the point of attachment to the tip of each spine; D: vertical distance from the tip on the 14th, 15th, and 16th hemal spines; L: total spine length. Significant differences between the sampling sites and reference site (S0) are indicated by an asterisk (p < 0.05).

**Figure 3:**
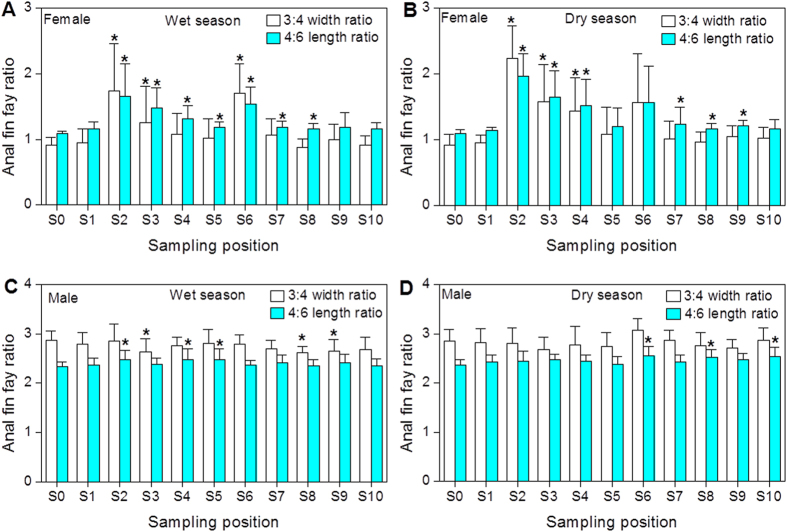
The morphological characteristics of anal fin rays for female and male mosquitofish form the Shima and Danshui Rivers as well as the reference site (S0). The length ratio of ray 4 and ray 6 (4/6L) and width ratio of ray 3 and ray 4 (3/4W) were used as the degree of the change of anal fins. Significant differences between sampling sites and reference site (S0) are indicated by an asterisk (p < 0.05).

In general, the changes of P/D ratios (P: perpendicular distance from the point of attachment of the hemal spine to its distal tip; D: perpendicular distance from the distal tip of the hemal spine to the center of the vertebral column) were more obvious than those of L/D ratios (L: total hemal spine length; D: perpendicular distance from the distal tip of the hemal spine to the center of the vertebral column), 3/4W ratios (width ratios of ray 3 and ray 4) and 4/6L ratios (length ratios of ray 4 and ray 6) in the collected mosquitofish from the study area used for the morphological analysis (Figs 2 and 3). Increased P/D ratios in female mosquitofish collected from the Shima River and Danshui River (except for S1) were observed when compared with those in the reference site (S0), and significant differences were found at almost all sampling sites (Fig. 2A,B). A majority of female mosquitofish from the sampling sites exhibited a masculinized anal fin and hemal spines (Figs 2A,B,E,F and 3A,B), suggesting masculinization of female mosquitofish in the rivers.

The P/D ratios were significantly decreased in male mosquitofish at the majority of sampling sites compared with the reference site (S0) (Fig. 2C,D). A majority of feminized male mosquitofish from the sampling sites exhibited a feminized gonopodium and hemal spines with female characteristics (Figs 2C,D,G,H and 3C,D), indicating feminization of male mosquitofish in the two rivers. In addition, ovotestes were also found in masculinized adult females from some sampling sites such as S2 and S6 by gonadal histopathology (Supplementary Fig. S2G and H).

### Reproduction-related gene expression in mosquitofish

**Figure 4:**
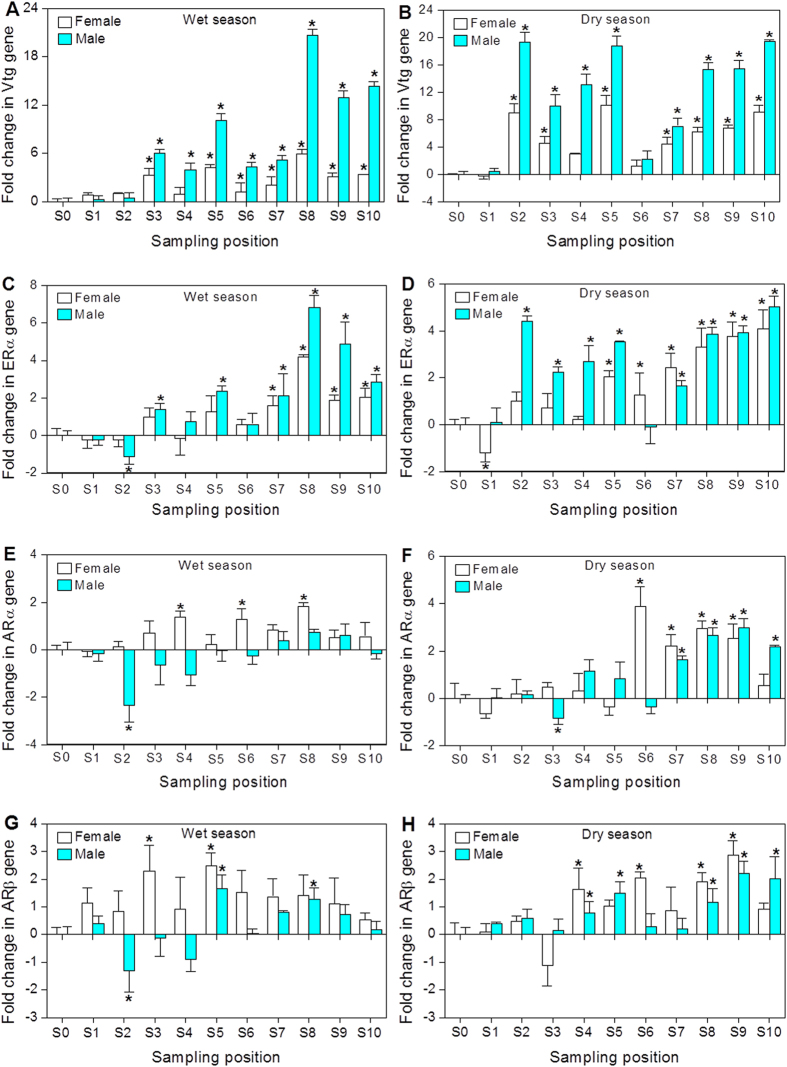
The mRNA expression level of Vtg, ERα, ARα, and ARβ˛mRNA in female and male mosquitofish from the Shima and Danshui Rivers as well as the reference site. Error bars represent the standard deviations of the measured values. The mRNA expression of each target gene was compared to that in the reference site (S0). Significant differences between the sampling sites and reference site (S0) are indicated by an asterisk (*p* < 0.05).

Hepatic Vtg mRNA expression in female and male mosquitofish was significantly elevated with a coincident significant increase in ERα mRNA expression at the majority of sampling sites compared to the reference site S0 (Fig. 4A–D). In general, induction of Vtg and ERα mRNA expression was more evident in the males than females at most sampling sites and periods. Significant induction in the relative mRNA expression of ARα and ARβ were detected at some sampling sites (Fig. 4E–H). Moreover, significant decreases in hepatic ARα and ARβ mRNA expression in male mosquitofish were also observed at sites S2 or S3 from the Shima River.

### Correlations between morphological and genetic responses in mosquitofish and hormonal activities in water samples

**Figure 5:**
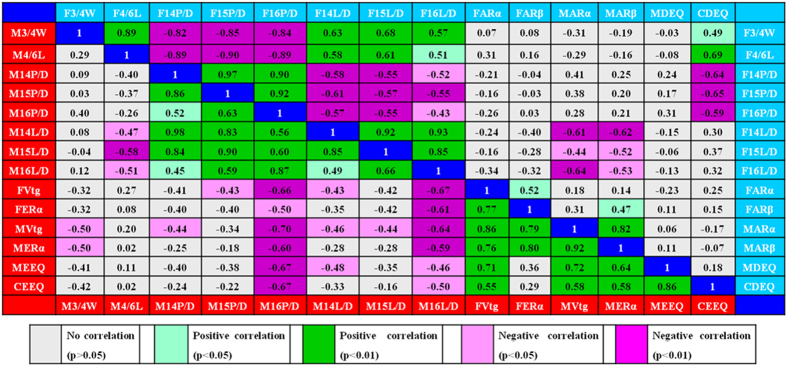
Correlations among the endpoints for the estrogenic and androgenic effects in mosquitofish, measured estrogenic and androgenic activities by *in vitro* bioassays, and calculated estrogenic and androgenic activities by chemical analysis. The numerical values in left of blue squares are Pearson correlation coefficients among the endpoints for the estrogenic effect in mosquitofish, measured estrogenic activity by *in vitro* bioassay, and calculated estrogenic activity by chemical analysis. The numerical values in right of blue squares are Pearson correlation coefficients among the endpoints for the androgenic effect in mosquitofish, measured androgenic activity by *in vitro* bioassay, and calculated androgenic activity by chemical analysis. F and M in front of the endpoints of mosquitofish represent female and male, respectively; MEEQ and CEEQ represent measured EEQ by YES and calculated EEQ by chemical analysis, respectively; MDEQ and CDEQ represent measured EEQ by YAS and calculated EEQ by chemical analysis, respectively.

Pearson correlation analysis was performed between morphological and genetic responses in mosquitofish and hormonal activities in water (Fig. 5). CEEQ had statistically significant correlations with Vtg and ERα mRNA expression in the males (*r* = 0.58, *p* < 0.01), and the ratio of 16P/D (P/D ratio of the 16th hemal spine) in the males (*r* = −0.67, *p* < 0.01), as well as the MEEQ (*r* = 0.86, *p* < 0.01). Like CEEQ, MEEQ was positively correlated with Vtg and ERα mRNA expression in the males (*r* = 0.64–0.72, *p* < 0.01), but negatively correlated with the ratio of 16P/D in the males (*r* = −0.67, *p* < 0.01). CDEQ showed significantly negative relationships with the ratios of 14P/D (*r* = −0.64, *p* < 0.01), 15P/D(*r* = −0.65, *p* < 0.01) and 16P/D (*r* = −0.59, *p* < 0.01) in the females. In addition, positive correlations were observed between the aqueous concentrations of the two alkylphenols and Vtg and ER gene expression levels, between the aqueous concentrations of some androgens (ADD, 17α-BOL, 17β-BOL, T and EADR) and fish morphological parameters and ARα and ARβ gene expression levels (Supplementary Table S3 and Supplementary Table S4).

**Figure 6:**
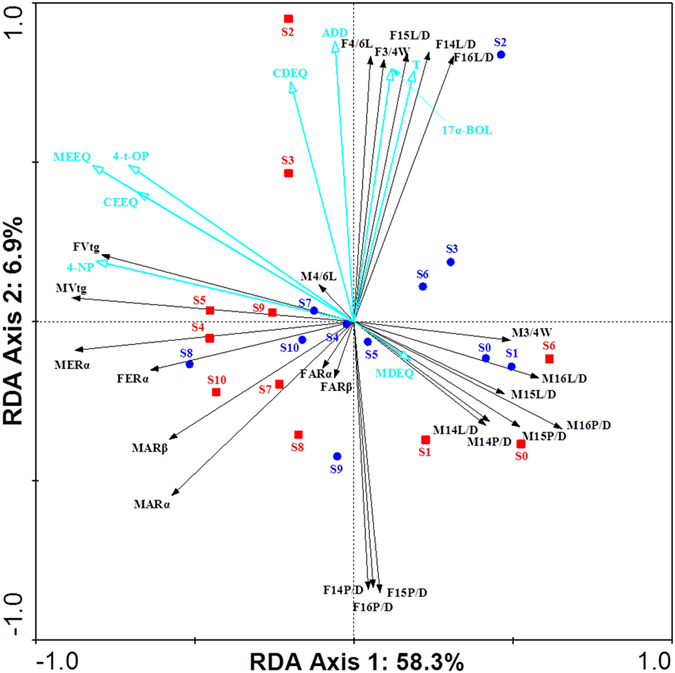
Redundancy analysis (RDA) ordination diagram (triplot) showing samples (blue circles for wet season, red squares for dry season), explanatory variables (green hollow arrows), and response variables (black solid arrows). First axis is horizontal, second axis is vertical. F and M in front of the endpoints of mosquitofish represent female and male, respectively; MEEQ and CEEQ represent measured EEQ by YES and calculated EEQ by chemical analysis, respectively; MDEQ and CDEQ represent measured EEQ by YAS and calculated EEQ by chemical analysis, respectively; BPA: bisphenol-A; 4-NP: 4-nonylphenols; ADD: androsta-1,4-diene-3,17-dione; AED: androsterone (ADR), 4-androstene-3,17-dione, EADR: epi-androsterone, T: testosterone. The angles among arrows denote the degree of correlation between the individual variables, and the smaller the angle, the larger the correlation. In addition, positively correlated variables are shown as arrows pointing in the same direction, negatively correlated variables pointing in opposite directions.

The results of redundancy analysis (RDA) for various measured data are displayed in Fig. 6. Some estrogenic and androgenic compounds were not included in the RDA analysis because they had high linear correlation with other compounds and showed high variance inflation factors (VIF > 10). The VIFs of the compounds chosen for RDA were reasonably low (from 1.4 to 7.7). The first ordination RDA axis (horizontal) was mainly correlated to MEEQ, CEEQ, 4-NP and 4-t-OP and explained 58.3% of the variation in the estrogenic and androgenic activities (82.8% of their relation to compounds). Vtg and ERα mRNA expression in the females and males as well as the morphological characteristics in the males were found to be strongly influenced by 4-NP and 4-t-OP. The second ordination axis (vertical), which was strongly associated with CDEQ, ADD, 17α-BOL and T, accounted for 6.9% of the variation (9.7% of their relation to compounds). ADD, 17α-BOL and T were found to have a major influence in the anal fin and hemal spines in the female mosquitofish. It should also be noted that both Pearson correlation analysis and RDA analysis showed many of the morphological traits were correlated with each other (Figs 5 and 6).

## Discussion

The present study made good use of both chemical analysis and bioassays to identify toxicants suspected of causing endocrine disrupting effects in fish. When the results of the morphological indices and reproduction-related gene mRNA expression levels in mosquitofish are taken together, there is sufficient evidence to suggest that mosquitofish in the Shima River and Danshui River experienced strong estrogenic and androgenic effects. The results from the present study also demonstrated that estrogens and androgens in the two rivers may be associated with the observed estrogenic and androgenic effects in mosquitofish.

Previous studies have shown shorter gonopodia (feminization) in male mosquitofish in response to exogenous estrogens^20^, and this is accompanied by a general delay in the development of hemal spines^14^,^19^. In the present study, male mosquitofish from the Shima River and Danshui River had a reduction in the development of gonopodia and hemal spines, as demonstrated by the decreased ratio of 4/6L, 3/4L 14P/D, 15P/D, or 16P/D. It should be noted that many of the morphological traits are correlated with each other. A similar result was also reported in our previous study, which showed estrogenic effects of male mosquitofish in the sewage-contaminated Hanxi River of South China based on the morphology of hemal spines^14^. It has been demonstrated that gonadal secretions are necessary for gonopodium development in male mosquitofish^21^. The observed shorter gonopodia in mosquitofish from two rivers in the present study indicates the alteration of endocrine function. In fact, delayed development of gonopodia and hemal spines in the males was also in accordance with the induction of Vtg mRNA expression in females and males at the study sites.

Previous studies about estrogenic effects have already demonstrated that the mRNA expression of hepatic Vtg can be induced in male fish exposed to estrogens, which is normally present at undetectable or low levels in males^15^,^22^. The results of the present study demonstrate that mosquitofish at the majority of sampling sites experienced transcriptional feminization as evidenced by the significant induction in Vtg and ERα mRNA expression. More importantly, Vtg mRNA expression levels in the males from some study sites reached or even exceeded those in females. High Vtg mRNA expression levels in mosquitofish can be associated with higher incidences of testicular malformation, higher amounts of oocyte malformation, lower sex steroid concentrations, and kidney dysfunction^23^. Given the relationship between Vtg and ERα, a consistent effect on both genes at most study sites of the present study is not surprising.

Concentrations of some estrogens and estrogenic activities (EEQ) measured in the present study showed strong correlations with those morphological and molecular parameters related to estrogenic effects in mosquitofish. Both Pearson correlation and RDA showed that estrogenic compounds such as NP and 4-t-OP in the rivers could have contributed to the induction of Vtg and ERα mRNA expression in mosquitofish. Previous laboratory studies have shown that NP and 4-t-OP have the potential to induce Vtg in males, form intersex gonads in males, and cause other signs of reproductive impairment^24^,^25^. In addition, NP and 4-t-OP have been reported to be the primary cause of feminized effects downstream of industrial wastewater discharges^26^. However, the contribution of natural and synthetic estrogens cannot be discarded since some of them were present in the sampling sites and others could be under our detection limit but they may still be high enough to contribute to estrogenic effects in fish.

Likewise, the measured *in vitro* estrogenic activities in the rivers of the present study support the linkage of estrogenic compounds to the observed morphological and genetic responses related to estrogenic effects in male mosquitofish. Therefore, those estrogenic compounds could be contributing to the observed estrogenic effects in mosquitofish in the rivers impacted by municipal wastewater.

The anal fins and hemal spines of female mosquitofish can serve as biomarker for androgen exposure, either in the laboratory or in the environment^13^,^14^. Female mosquitofish from the Shima River and Danshui River were found to have masculinized anal fin and hemal spines at almost all sampling sites, despite no significant morphological changes in a few sites when compared to the reference site. It seems that the hemal spines in the females were more sensitive as morphological biomarkers than the anal fin rays. In addition, ovotestes were also found in masculinized adult females from some sampling sites. More interestingly, masculinized female mosquitofish showed significant increase in Vtg and ERα mRNA expression at some sampling sites such as S2 andS3, suggesting the simultaneous presence of super-feminization at the transcriptional level in masculinized female mosquitofish. To date, however, the formation mechanism of this phenomenon is still not known. According to the RDA and Pearson correlation analysis, androgens such as ADD, T, and EADR could be responsible for morphological masculinization in the females. The morphological biomarkers were also found to be better correlated to the CDEQ than MDEQ values. This could be attributed to matrix interferences often experienced during *in vitro* YAS bioassay and other factors as reported by others^17^,^27^. Thus we should be cautious to use *in vitro* YAS bioassay data to assess androgenic effects in fish^28^.

The action of androgens is classically mediated by androgen receptors (ARs), which act as ligand-dependent transcription factors, controlling the transcription of target genes. Therefore, any disruption in the signaling of ARs may lead to impairment of genomic pathways and downstream processes^15^. Changes in ARα and ARβ mRNA expression levels in mosquitofish from the study sites did not have a consistent pattern with an increase, decrease, or no change, which may be attributed to a complex feedback of the AR-mediated gene expression in fish^1^,^29^. The differential expression currently observed in ARα and ARβ mRNA expression in the female mosquitofish within different sampling sites was further reflected by the weak correlations to the calculated and measured DEQ values in the rivers. This may suggest the presence of other androgenic compounds in the rivers; but more likely this may suggest that ARs are not good biomarkers for mosquitofish exposed to androgens as found in previous studies^15^,^29^. Although it is still unclear whether these transcription-level effects of ARs in mosquitofish as a consequence of exposure to municipal wastewater are translated into morphology-level abnormalities, abnormal AR mRNA expression levels in wild mosquitofish are undeniably a dysfunctional AR signaling.

In summary, the findings in this study showed strong estrogenic and androgenic effects co-occurring in mosquitofish from the Shima River and Danshui River, and good correlations between the analyzed EDCs levels (and hormonal activities) in the rivers and the morphological effects observed in mosquitofish. Since these chemicals are mainly derived from municipal wastewaters, the discharge of municipal wastewaters into the rivers is sufficient to cause estrogenic and androgenic effects in mosquitofish, resulting in feminization of male mosquitofish and masculinization of female mosquitofish. Toxicity identification evaluation (TIE) and effects directed assay (EDA) should be performed in future studies to identify specific toxicants causing endocrine disrupting effects. Further research is also needed to explore the population level effects in mosquitofish in rivers impacted by municipal wastewaters.

## Methods

### Study area

Shima River and Danshui River were selected as the study areas since they are located in a rapidly urbanized Pearl River Delta region of South China (Fig. 1). Among the 10 selected sampling sites, 5 sites were on the Shima River (S1-S5), and 5 sites on the Danshui River (S6-S10), with S1 used as the reference site as it is located in the upstream section of the Shima River with little human activity. Another reference site (S0) was also included, and it is located in the upstream of the Liuxi River in the region with little human activity. Both reference sites are located at the outlet of reservoirs.

### Fish sampling

Mosquitofish (*Gambusia affinis)* were captured by electrofishing and/or netting from the selected sites in July 2012 (wet season) and December 2012 (dry season). Surface water samples were also collected simultaneously with the fish. During the sampling, water quality parameters were measured according to standard methods^30^, and these data can be found in Supplementary Table S1. All the collected fish were kept alive in plastic storage boxes filled with river water from their respective collection sites and aerated to ensure sufficient oxygen supply. Once in the laboratory, fish were sorted into sexually mature females, immature females, gravid females, mature males, and immature males. The criteria to determine whether fish are mature were based on previous studies^13^,^14^. In order to avoid sampling bias, only mature females and males from each site were randomly selected for further measurement. A total of 1157 female mosquitofish and 1143 male mosquitofish were used for standard length and body weight. The numbers, mean standard length and mean body weight of mosquitofish in each site can be found in Supplementary Table S2. The bodies of a total of 501 females and 496 males from all sites (20–27 of each sex at each site) were preserved in 70% ethanol for determination of morphology. The livers of 15 females and 15 males from each site were preserved in RNAlater (Sigma) for determination of target gene expression. All procedures with fish described in this study were performed in accordance with the 2004 Guidelines (Use of Fishes in Research 2004)^31^. They were also approved by the Animal Care and Use Committee of South China Agricultural University.

### Water sample collection and extraction

Surface water samples were collected in 1 L amber glass bottles from each site. The collected water samples were transported in coolers to the laboratory and stored at 4 °C, and then processed within 48 h. Six replicates of the surface water samples were collected from each site. Three replicates were used for YES and YAS bioassays, whereas the other three replicates were spiked with the internal standards for chemical analysis of estrogens and androgens. The water samples for YES and YAS bioassays were extracted by HLB cartridges (Waters Oasis 6 mL, 500 mg) according to the method reported by Zhao *et al.*^32^. The target estrogenic and androgenic compounds in water samples were extracted using solid phase extraction according to our previous method^5^.

### Chemical analysis

Twenty-one target compounds were analysed: 4 estrogens (diethylstilbestrol (DES), estrone (E1), 17β-estradiol (E2), and 17α-ethynyl estradiol (EE2)), 3 xenoestrogens (bisphenol-A (BPA), 4-nonylphenols (4-NP), and 4-t-octylphenol (4-t-OP)), 14 androgens (androsta-1,4-diene-3,17-dione (ADD), androsterone (ADR), 4-androstene-3,17-dione (AED), 17α-boldenone (17α-BOL), 17β-boldenone (17β-BOL), 5α-dihydrotestosterone (5α-DHT), epi-androsterone (EADR), 4-hydroxy-androst-4-ene-17-dione (4-OHA), methyltestosterone (MT), 19-nortestoserone (19-NT), testosterone (T), 17α-trenbolone (17α-TBL), 17β-trenbolone (17β-TBL), and Stanozolol (S)).

A pentafluorobenzoyl chloride derivatization method was applied to the quantification of estrogens in the extracts prior to analysis by gas chromatography–mass spectrometry with negative chemical ionization (Agilent 6890N/5975B, USA) as described by Zhao *et al.*^32^. The androgens in the extracts of water samples were measured by rapid resolution liquid chromatography–electrospray ionization tandem mass spectrometry (Agilent 1200 LC-Agilent 6460, USA), and its detailed operating method is detailed in the previous study^5^. All data obtained from the analysis were subject to strict quality assurance and quality control (QA/QC) procedures. The limits of detection and quantification are given in Table 1.

### *In vitro* bioassays

The YES and YAS bioassays were based on the methods as described by Zhao *et al.*^33^. Methanol was used as the blank control; E2 was used as the positive control of YES with an initial concentration of 0.2 μM; and DHT was used as the positive control of YAS with an initial concentration of 2 μM. The levels of estrogenic and androgenic activities were then expressed as E2 equivalent (EEQ), and DHT equivalent (DEQ), respectively.

### Characterization of basic fish parameters and morphology

Fish were killed in an ice bath, and their standard length (from the snout to beginning of the caudal fin) and body weight were measured. The method for preparation of the anal fin and skeletons is described by Xie *et al.*^14^. Briefly, preserved mosquitofish were soaked in purified water to rehydrate and then placed in 1% potassium hydroxide to remove the soft tissue. The anal fin and skeletons of mosquitofish were photographed using a camera mounted on a stereomicroscope.

The length ratio of ray 4 and ray 6 (4/6L) and width ratio of ray 3 and ray 4 (3/4W) were used to assess impacts to anal fin development. Morphological measurements of hemal spines were performed according to the method developed by Rawson *et al.*^19^. These measurements included total spine length (L), perpendicular distance from the point of attachment of the hemal spine to its distal tip (P, positive where the spines were anteriorly directed and negative where the spines were posteriorly directed), and perpendicular distance from the distal tip of the hemal spine to the center of the vertebral column (D) on the 14th, 15th and 16th hemal spines (labeled as 14L, 15L and 16L; 14P, 15P and 16P; and 14D, 15D and 16D) (Supplementary Fig. S1). In the present study, the ratios of 14P/D, 15P/D, 16P/D, 14L/D, 15L/D and 16L/D were used to assess the elongation and the anterior bending of the hemal spines on the 14th, 15th and 16th vertebrae in mosquitofish.

### The mRNA expression of reproduction-related genes in mosquitofish

Total RNA was extracted from the pooled livers of five female or male fish at each sampling site (three replicate pooled liver samples per site) using Trizol reagent (Invitrogen) according to the manufacturer’s instructions. First-strand cDNA synthesis was performed with 1 μg of total RNA using ReverTra Ace® qPCR RT Master Mix with gDNA Remover (Toyobo, Japan) in a total volume of 20 μL according to the manufacturer’s instructions.

The qPCR analysis was performed on the Applied Biosystems ViiA™ 7 Dx (ABI) using the THUNDERBIRD SYBR®qPCRMix (Toyobo), in a final volume of 20 μL. Primer sequences for *G. affinis* Vtg, ERα, ARα, ARβ and β-Actin were obtained from Huang *et al.*^15^. The *G. affinis* ribosomal protein L8 (RPL8) gene sequence was generated using next generation RNA sequencing (GenBank, accession number DQ865277) as source information: forward 5′-CGAGGGAACCATCATCTGCT-3′and reverse 5′-AGATGACGGTGGCGTAGTTTC-3′. The geometric mean of measured transcript abundance for β-Actin and RPL8 were used to normalize the mRNA expression of four target genes (Vtg, ERα, ARα, and ARβ). Relative mRNA expression was calculated with the 2^−ΔΔCt^ method^34^.

### Statistical analyses

Data on morphological and genetic responses in mosquitofish are presented as mean ± standard deviation (SD) in each site. All physiological endpoints and gene expression patterns were assessed for normality and homogeneity of variances using Kolmogorov-Smirnov and Levene’s tests, respectively. Raw data were log transformed to meet the assumptions of one-way analysis of variance (ANOVA). Statistical differences between sampling sites for morphological and genetic responses in mosquitofish were determined by ANOVA, followed by Dunnett (homogeneous variance) or Dunnett’s T3 (heterogeneous variance). Differences were considered statistically significant when p < 0.05. Correlations between morphological or genetic biomarkers in mosquitofish and hormonal activities in water were analyzed using Pearson correlation and redundancy analysis (RDA). For Pearson correlation analysis, the data were log transformed to render them more nearly normal. RDA analysis was selected according to an initial detrended correspondence analysis (DCA). DCA revealed that the data of the estrogenic and androgenic responses exhibited a linear, rather than a unimodal, response to estrogens and androgens, thus RDA as a linear constrained ordination method was chosen for data analysis. In RDA, the estrogenic and androgenic responses were used as “response variables”, and the ordination axes were constrained to be linear combinations of the estrogenic and androgenic compounds (explanatory variables). All data applied in RDA were log transformed. The Monte Carlo permutation test (499 permutations) was performed to determine the significance of the estrogenic and androgenic compounds in accounting for the variance of the estrogenic and androgenic responses by assessing p-values. All data analysis was performed with software SPSS 13.0, Origin 7.5 and Canoco 4.5 for Windows.

## Additional Information

**How to cite this article**: Huang, G.-Y. *et al.* Feminization and masculinization of western mosquitofish (*Gambusia affinis*) observed in rivers impacted by municipal wastewaters. *Sci. Rep.* **6**, 20884; doi: 10.1038/srep20884 (2016).

## Supplementary Material

Supporting Information

## Figures and Tables

**Table 1:** Concentrations of 21 endocrine disrupting chemicals by chemical analysis and the estrogenic and androgenic activities measured by *in vitro* bioassays in the Shima and Danshui Rivers and the reference sites.

Compounds^a^	Reference sites^b^				Shima River^b^				Danshui River^b^				LOD^c^	LOQ^c^
	Range	Mean	Median	Freq	Range	Mean	Median	Freq	Range	Mean	Median	Freq		
Estrogens (ng/L)														
Diethylstilbestrol	ND				ND-22.0	5.04	0.25	38	ND				0.20	0.50
Estrone	ND-18.3	5.06	0.87	50	ND-32.0	11.5	10.5	25	ND-23.6	8.19	5.85	80	0.20	0.50
17β-estradiol	ND-1.01	0.63	0.50	25	ND-3.70	1.92	1.77	63	ND-2.78	1.06	0.5	40	0.30	1.00
EE2	ND				ND				ND				0.20	0.70
Bisphenol-A	5.21–815	247	84	100	276–28900	9870	1610	100	32.3–5770	1560	906	100	0.70	2.00
4-nonylphenols	106–2840	861	251	100	1210–7450	3710	3040	100	93.9–10900	4360	3450	100	2.00	7.00
4-t-octylphenol	2.37–14.8	8.64	8.69	100	17.7–408	178	148	100	1.39–188	61.1	21.0	100	0.30	1.00
CEEQ	0.09–7.36	2.31	0.89	100	1.21–16.0	8.76	10.6	100	0.06–13.2	6.17	5.94	100		
MEEQ	0.2–0.78	0.38	0.28	100	2.67–82.6	26.1	20.6	100	ND-29.2	11.4	8.69	90		0.2
Androgens (ng/L)														
ADD	ND-1.88	1.02	1.10	75	3.00–166	31.4	6.28	100	ND-12.1	3.66	3.26	90	0.08	0.28
Androsterone	ND				ND-16.6	2.65	0.67	13	ND-12.5	1.84	0.67	10	0.40	1.33
AED	ND-1.42	0.74	0.69	50	2.20–16.3	5.66	3.95	100	1.27–11.2	4.65	2.73	100	0.11	0.37
17α-boldenone	ND-2.25	0.70	0.19	25	ND-5.25	1.49	0.19	38	ND				0.11	0.38
17β-boldenone	ND-1.21	0.44	0.19	25	ND-10.5	3.12	1.37	75	ND-1.71	0.63	0.19	40	0.11	0.38
5α-DHT	ND				ND				ND-6.69	1.25	0.65	10	0.39	1.30
Epi-androsterone	ND				8.40–64.2	24.6	14.7	100	ND-17.7	4.20	0.44	30	0.26	0.87
4-OHA	ND				ND				ND				0.17	0.56
Methyltestosterone	ND				ND				ND				0.07	0.24
19-nortestoserone	ND				ND				ND				0.31	1.03
Testosterone	ND				ND-1.98	0.58	3.95	25	ND-1.02	0.27	0.19	10	0.11	0.37
17α-trenbolone	ND				ND				ND				0.09	0.31
17β-trenbolone	ND				ND-16.6	2.30	0.25	13	ND				0.15	0.50
Stanozolol	ND				ND				ND				0.01	0.02
CDEQ	ND-4.55	2.16	2.05	75	ND-278	68.0	28.0	88	2.89–39.0	14.7	8.59	100		
MDEQ	ND-26.9	11.8	9.52	50	ND-46.0	9.36	1.25	25	ND-34.7	6.71	2.89	50		2.5

^a^EE2, 17α-ethynylestradiol; ADD, Androsta-1,4-diene-3,17-dione; AED, 4-androstene-3,17-dione; 5α-DHT, 5α-dihydrotestosterone; 4-OHA,4-hydroxy-androst-4-ene-17-dione; MEEQ and CEEQ represent measured EEQ by YES and calculated EEQ by chemical analysis, respectively; MDEQ and CDEQ represent measured EEQ by YAS and calculated EEQ by chemical analysis, respectively.

^b^Mean value and median value are calculated using a statistical method to estimate the concentration of each compound in the non-detect samples (1/2 LOQ). Frequency is calculated based on those with concentrations higher than the limit of quantification. Freq, the detection frequency. ND, not determined.

^c^LOD: limit of detection; LOQ: limit of quantitation.
