# Supplementary material for: Feminization and masculinization of western mosquitofish (Gambusia affinis) observed in rivers impacted by municipal wastewaters
Source: Sci Rep. 2016 Feb 15;6:20884. doi: 10.1038/srep20884 (PMC4753483; doi:10.1038/srep20884)
Supplement: Supporting Information [file srep20884-s1.doc]

**Supplementary information**

**Feminization and masculinization of western mosquitofish (*Gambusia affinis*) observed in rivers impacted by municipal wastewaters**

Guo-Yong Huang, You-Sheng Liu, Xiao-Wen Chen, Yan-Qiu Liang, Shuang-Shuang Liu, Yuan-Yuan Yang, Li-Xin Hu, Wen-Jun Shi, Fei Tian, Jian-Liang Zhao, Jun Chen, Guang-Guo Ying*

State Key Laboratory of Organic Geochemistry, CAS Centre for Pearl River Delta Environmental Pollution and Control Research, Guangzhou Institute of Geochemistry, Chinese Academy of Sciences, Guangzhou 510640, China

*Corresponding author (GG Ying)

Tel/Fax: (8620) 85290200

E-mail address: guangguo.ying@gmail.com; guang-guo.ying@gig.ac.cn

**This file includes the following tables and figures:**

**Table S1.** Water quality parameters of sampling sites in the Shima and Danshui Rivers

**Table S2.** The basic parameters of mosquitofish collected from the Shima and Danshui Rivers

**Table S3.** Correlation between the estrogenic effect in mosquitofish and estrogenic activity in water samples by Pearson correlation analysis

**Table S4.** Correlation between the androgenic effect in mosquitofish and androgenic activity in water samples by Pearson correlation analysis

**Figure S1.** The anal fin and hemal spines of female (A, C and E) and male (B, D, and F) mosquitofish. Total length of anal fin ray was calculated as the sum of a, b and c for 4 anal fin ray and 6 anal fin ray (A and B). The widths of ray 3 and ray 4 were measured at the point where ray 4 bifurcates (labeled as 3W and 4W) (C and D). Total length of the hemal spine was calculated as the sum of a, b and c for each spine; perpendicular distance from the point of attachment to the tip of each spine (labeled as 14P, 15P and 16P); depth measured from the distal tip of the spine to the vertebral column(labeled as 14D, 15D and 16D) (E and F).

**Figure S2**. Pictures of anal fin and gonadal histology for masculinized female mosquitofish. Gonadal structures were identified based on Leusch et al. (2006): Primary oocytes (O1), cortical alveoli oocytes (O2), spermatozeugmata (Sz), spermatocytes (S1).

**Table S1.** Water quality parameters of sampling sites in the Shima and Danshui Rivers

| Sites | Geographic location (N, E) | | River | pH | | DO (mg/L) | | COD (mg/L) | | BOD5 (mg/L) | | TP (mg/L) | | TN (mg/L) | | NH_4_-N (mg/L) | |
| --- | --- | --- | --- | --- | --- | --- | --- | --- | --- | --- | --- | --- | --- | --- | --- | --- | --- |
|  |  |  |  | Wet | Dry | Wet | Dry | Wet | Dry | Wet | Dry | Wet | Dry | Wet | Dry | Wet | Dry |
| S0 | 114°14’38” | 22°05’37” | Liuxi River | 6.91 | 7.3 | 6.32 | 4.56 | 51.0 | 8.11 | 1.91 | 2.10 | 0.05 | 0.08 | 2.46 | 3.94 | 0.41 | 1.28 |
| S1 | 114°06’45” | 22°50’30” | Shima River | 7.09 | 6.64 | 3.32 | 2.68 | 17.6 | 49.3 | 0.68 | 6.01 | 0.05 | 0.01 | 4.79 | 0.75 | 0.82 | 0.27 |
| S2 | 114°06’52” | 22°47’10” | Shima River | 6.82 | 6.75 | 0.83 | 2.25 | 76.2 | 243 | 4.97 | 11.6 | 1.78 | 2.88 | 11.3 | 13.7 | 6.84 | 7.11 |
| S3 | 114°12’27” | 22°49’54” | Shima River | 7.21 | 6.72 | 3.30 | 3.76 | 12.9 | 98.5 | 1.11 | 14.3 | 0.62 | 1.93 | 7.25 | 9.71 | 0.89 | 8.48 |
| S4 | 114°07’53” | 22°50’57” | Shima River | 6.94 | 6.64 | 0.79 | 2.13 | 23.4 | 217 | 0.91 | 12.8 | 0.48 | 1.77 | 9.85 | 12.6 | 2.53 | 8.48 |
| S5 | 114°06’39” | 23°01’11” | Shima River | 7.04 | 6.50 | 2.20 | 1.12 | 58.6 | 158 | 0.87 | 9.38 | 0.47 | 1.01 | 8.34 | 11.9 | 2.53 | 8.34 |
| S6 | 114°05’17” | 23°03’34” | Danshui River | 6.82 | 6.94 | 6.05 | 4.02 | 9.96 | 4.05 | 0.80 | 3.21 | 0.05 | 0.01 | 3.69 | 0.89 | 0.41 | 0.14 |
| S7 | 114°15’27” | 22°43’53” | Danshui River | 7.00 | 6.43 | 2.59 | 6.30 | 18.8 | 16.2 | 1.39 | 1.18 | 0.38 | 0.46 | 11.5 | 9.30 | 0.48 | 1.37 |
| S8 | 114°24’52” | 22°48’11” | Danshui River | 6.77 | 6.49 | 1.63 | 3.44 | 32.2 | 16.2 | 2.19 | 8.22 | 0.88 | 0.40 | 11.3 | 9.03 | 6.84 | 6.09 |
| S9 | 114°27’13” | 22°47’59” | Danshui River | 7.03 | 6.83 | 4.30 | 3.00 | 10.5 | 12.2 | 0.99 | 1.10 | 0.35 | 0.33 | 6.84 | 10.1 | 2.39 | 5.54 |
| S10 | 114°29’28” | 22°56’38” | Danshui River | 7.25 | 6.74 | 1.36 | 1.62 | 14.7 | 21.9 | 0.72 | 1.28 | 0.25 | 0.19 | 5.40 | 9.44 | 2.05 | 6.15 |

**Table S2.** The basic parameters of mosquitofish collected from the Shima and Danshui Rivers

|  | Females | | | | | | |  | Males | | | | | | |
| --- | --- | --- | --- | --- | --- | --- | --- | --- | --- | --- | --- | --- | --- | --- | --- |
|  | Wet | | |  | Dry | | |  | Wet | | |  | Dry | | |
| Sites | *n*† | Standard length (mm) | Body weight  (mg) |  | *n* | Standard length (mm) | Body weight (mg) |  | *n* | Standard length  (mm) | Body weight (mg) |  | *n* | Standard length  (mm) | Body weight  (mg) |
| S0 | 60 | 27.3±3.68§ | 482±208 |  | 90 | 25.8±3.06 | 359±126 |  | 60 | 18.1±1.43 | 96.5±25.2 |  | 82 | 20.7±1.96 | 153±48.1 |
| S1 | 60 | 25.5±4.05* | 359±194* |  | 31 | 25.6±3.51 | 262±110* |  | 60 | 17.7±1.10 | 77.2±14.1* |  | 26 | 19.8±2.29 | 97.6±24.5* |
| S2 | 32 | 22.6±2.99* | 232±96.0* |  | 26 | 22.0±4.69* | 202±124* |  | 60 | 17.2±1.47* | 83.0±20.4* |  | 30 | 19.9±2.99 | 128±58.4 |
| S3 | 60 | 21.2±4.40* | 205±116* |  | 24 | 25.1±3.02 | 290±121* |  | 60 | 17.2±1.77* | 81.5±26.0* |  | 21 | 22.0±3.3*6 | 177±82.7 |
| S4 | 60 | 22.2±3.59* | 240±133* |  | 60 | 21.9±4.39* | 214±119* |  | 60 | 17.8±1.50 | 87.2±22.7* |  | 59 | 18.0±1.64* | 104±28.4* |
| S5 | 60 | 22.2±3.27* | 215±95.6* |  | 17 | 22.3±5.62* | 224±161* |  | 60 | 17.6±1.43 | 82.3±17.9* |  | 23 | 18.7±1.38* | 103±24.2* |
| S6 | 62 | 28.3±2.75 | 407±141* |  | 102 | 23.9±3.59* | 218±99.5* |  | 60 | 19.1±1.42* | 94.7±24.3 |  | 56 | 19.3±2.37* | 95.4±20.1* |
| S7 | 62 | 25.0±3.80* | 386±220* |  | 41 | 23.6±3.99* | 274±144* |  | 60 | 17.5±1.44* | 84.1±22.2* |  | 42 | 17.9±1.82* | 112±139* |
| S8 | 56 | 19.1±3.94* | 154±107* |  | 43 | 24.9±3.89 | 317±158 |  | 62 | 17.2±1.47* | 80.4±22.2* |  | 44 | 20.3±2.94 | 138±61.7 |
| S9 | 64 | 21.1±2.41* | 189±76.0* |  | 47 | 24.6±2.93 | 274±90.4* |  | 60 | 16.5±1.36* | 68.1±18.1* |  | 50 | 17.4±1.14* | 76.7±16.3* |
| S10 | 60 | 19.6±2.64* | 145±73.7* |  | 40 | 22.0±2.77* | 205±70.7* |  | 50 | 16.2±1.55* | 68.7±17.7* |  | 58 | 18.9±2.62* | 119±46.4* |

† represents numbers of mosquitofish used for the measurement of the basic parameters in each sampling site.

* represents the significant differences between sampling sites and reference site (S0) (*p*<0.05).

§Data are presented as means ± standard deviation (SD).

**Table S3.** Correlation between the estrogenic effect on mosquitofish and estrogenic activity in water samples by Pearson correlation analysis

|  | 4-t-OP | NP | BPA | E1 | E2 | DES | CEEQ | MEEQ |
| --- | --- | --- | --- | --- | --- | --- | --- | --- |
| M3/4W | -0.34 | -0.55** | -0.27 | -0.25 | -0.10 | -0.11 | -0.42 | -0.41 |
| M4/6L | 0.18 | -0.02 | 0.26 | -0.10 | 0.18 | 0.08 | 0.02 | 0.11 |
| M14P/D | -0.25 | -0.21 | -0.36 | -0.20 | -0.24 | -0.07 | -0.24 | -0.40 |
| M15P/D | -0.23 | -0.17 | -0.35 | -0.13 | -0.23 | -0.17 | -0.22 | -0.38 |
| M16P/D | -0.59** | -0.54** | -0.55** | -0.45* | -0.50* | -0.30 | -0.67** | -0.67** |
| M14L/D | -0.34 | -0.26 | -0.43* | -0.25 | -0.31 | -0.17 | -0.33 | -0.48* |
| M15L/D | -0.19 | -0.16 | -0.34 | -0.01 | -0.25 | -0.29 | -0.16 | -0.35 |
| M16L/D | -0.48* | -0.53* | -0.50* | -0.30 | -0.36 | -0.20 | -0.50* | -0.46* |
| FVtg | 0.64** | 0.64** | 0.56* | 0.40 | 0.47* | -0.10 | 0.55** | 0.71** |
| FERα | 0.23 | 0.56** | 0.25 | 0.25 | 0.16 | -0.16 | 0.29 | 0.36 |
| MVtg | 0.60** | 0.69** | 0.54** | 0.36 | 0.43* | 0.09 | 0.58** | 0.72** |
| MERα | 0.53* | 0.75** | 0.50 | 0.40 | 0.31 | 0.03 | 0.58** | 0.64** |

* Pearson correlation is significant at the 0.05 level (2-tailed). ** Pearson correlation is significant at the 0.01 level (2-tailed). F and M in front of the endpoints of mosquitofish represent female and male, respectively. CEEQ and MEEQ represent calculated EEQ by chemical analysis and measured EEQ by YES, respectively. 4-t-OP: 4-t-octylphenol; 4-NP: 4-nonylphenols; BPA: bisphenol-A; E1: estrone; E2: 17β-estradiol; DES: diethylstilbestrol.

**Table S4.** Correlation between the androgenic effect on mosquitofish and androgenic activity in water samples by Pearson correlation analysis

|  | ADD | 17α-BOL | 17β-BOL | AED | T | EADR | ADR | 5α-DHT | 17β-TBL | CDEQ | MDEQ |
| --- | --- | --- | --- | --- | --- | --- | --- | --- | --- | --- | --- |
| F3/4W | 0.61** | 0.52* | 0.34 | 0.44* | 0.63** | 0.52* | -0.05 | 0.32 | 0.27 | 0.49* | -0.03 |
| F4/6L | 0.70** | 0.53** | 0.45* | 0.55** | 0.59** | 0.67** | 0.21 | 0.19 | 0.15 | 0.69** | -0.08 |
| F14P/D | -0.64** | -0.53* | -0.56* | -0.47* | -0.61** | -0.61** | -0.21 | -0.23 | -0.19 | -0.64** | 0.24 |
| F15P/D | -0.70* | -0.49* | -0.58** | -0.49* | -0.61** | -0.64** | -0.21 | -0.23 | -0.19 | -0.65** | 0.17 |
| F16P/D | -0.59* | -0.41 | -0.45* | -0.49* | -0.55** | -0.59** | -0.11 | -0.27 | -0.15 | -0.59** | 0.31 |
| F14L/D | 0.29 | 0.62** | 0.44* | -0.03 | 0.56** | 0.40 | 0.18 | 0.12 | 0.15 | 0.30 | -0.15 |
| F15L/D | 0.30 | 0.61** | 0.38 | 0.08 | 0.57** | 0.45* | 0.13 | 0.15 | 0.23 | 0.37 | -0.06 |
| F16L/D | 0.38 | 0.66** | 0.53* | -0.07 | 0.53* | 0.50* | 0.21 | 0.07 | 0.12 | 0.32 | -0.13 |
| FARα | -0.01 | -0.30 | -0.27 | 0.29 | -0.13 | -0.03 | -0.01 | 0.12 | -0.07 | 0.25 | -0.23 |
| FARβ | 0.12 | -0.39 | -0.23 | 0.20 | -0.14 | 0.01 | -0.04 | 0.12 | 0.15 | 0.15 | 0.11 |
| MARα | -0.13 | -0.38 | -0.42 | 0.26 | -0.38 | -0.35 | -0.28 | -0.15 | 0.19 | -0.17 | 0.06 |
| MARβ | 0.03 | -0.28 | -0.24 | 0.25 | -0.38 | -0.15 | -0.24 | -0.15 | 0.09 | -0.07 | 0.11 |

* Pearson correlation is significant at the 0.05 level (2-tailed). ** Pearson correlation is significant at the 0.01 level (2-tailed). F and M in front of the endpoints of mosquitofish represent female and male, respectively. MDEQ and CDEQ represent measured DEQ by YAS and calculated DEQ by chemical analysis, respectively. ADD: androsta-1,4-diene-3,17-dione; 7α-BOL: 17α-boldenone; 17β-BOL: 17β-boldenone; AED: 4-androstene-3,17-dione; T: testosterone; EADR: epi-androsterone; ADR: androsterone; 5α-DHT: 5α-dihydrotestosterone; 17β-TBL: 17β-trenbolone.

**Figure S1**

**
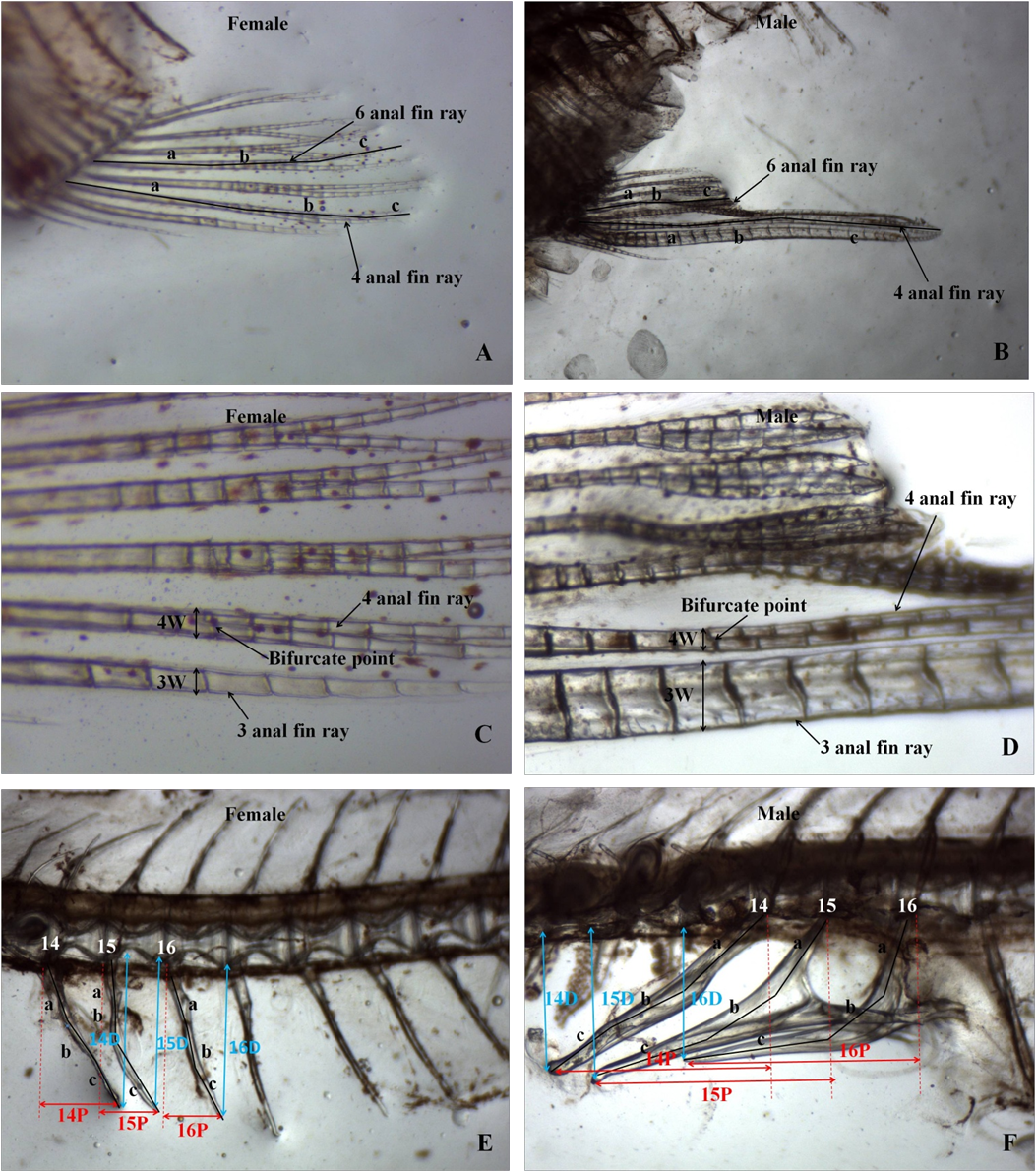
**

**Figure S1.** The anal fin and hemal spines of female (A, C and E) and male (B, D, and F) mosquitofish. Total length of anal fin ray was calculated as the sum of a, b and c for 4 anal fin ray and 6 anal fin ray (A and B). The widths of ray 3 and ray 4 were measured at the point where ray 4 bifurcates (labeled as 3W and 4W) (C and D). Total length of the hemal spine was calculated as the sum of a, b and c for each spine; perpendicular distance from the point of attachment to the tip of each spine (labeled as 14P, 15P and 16P); depth measured from the distal tip of the spine to the vertebral column(labeled as 14D, 15D and 16D) (E and F).

**Figure S2**


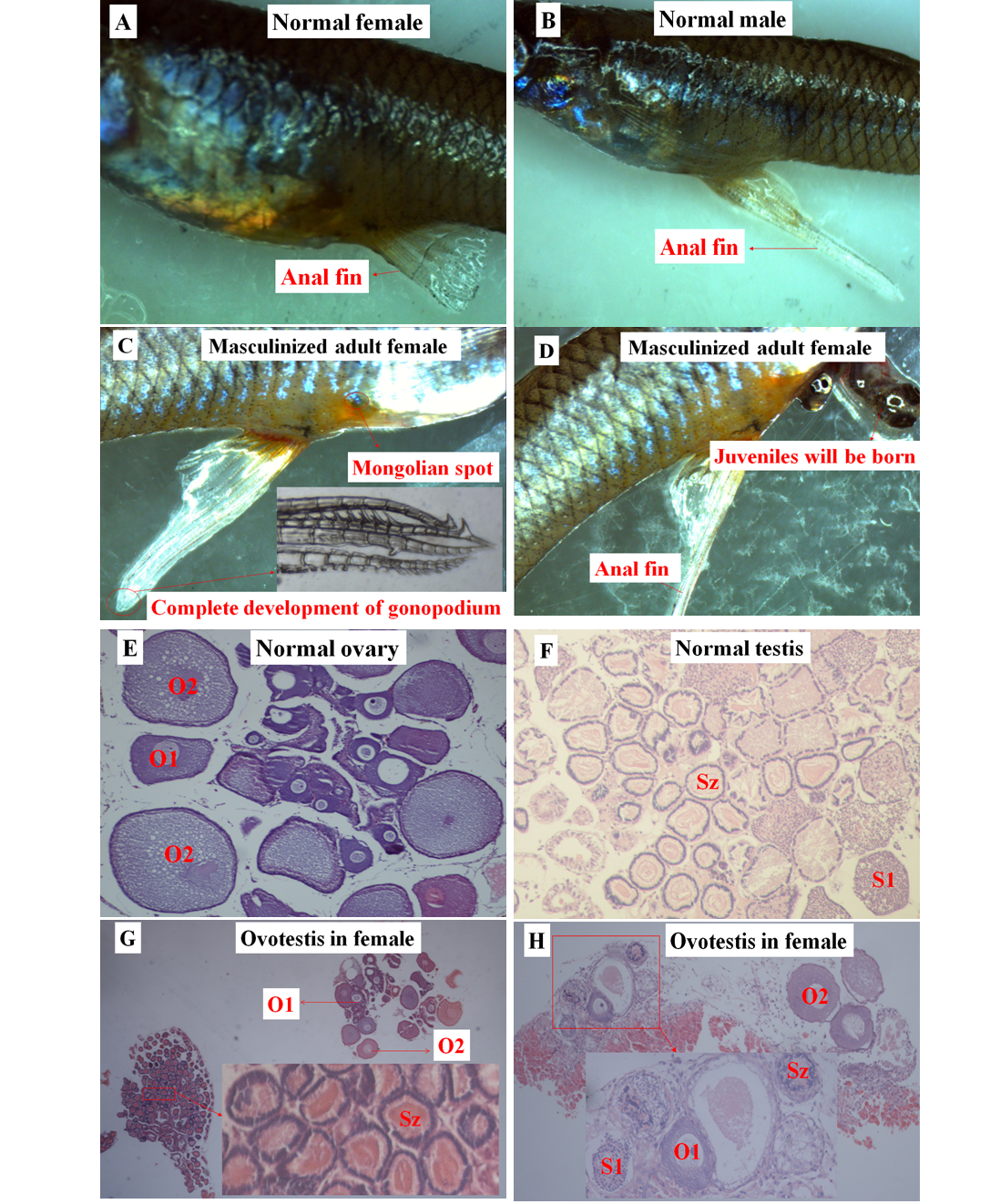


**Figure S2**. Pictures of anal fin and gonadal histology for masculinized female mosquitofish. Gonadal structures were identified based on Leusch et al. (2006): Primary oocytes (O1), cortical alveoli oocytes (O2), spermatozeugmata (Sz), spermatocytes (S1).

References:

Leusch FDL, Chapman HF, Kay GW, Gooneratne SR, Tremblay LA. 2006. Anal fin morphology and gonadal histopathology in mosquitofish (*Gambusia holbrooki*) exposed to treated municipal sewage effluent. Arch Environ Contam Toxicol 50:562-574.
